# Distribution and Molecular Characterization of β-Glucans from Hull-Less Barley Bran, Shorts and Flour

**DOI:** 10.3390/ijms12031563

**Published:** 2011-02-28

**Authors:** Xueling Zheng, Limin Li, Qi Wang

**Affiliations:** 1 Grain College, Henan University of Technology, Zhengzhou, 450052, China; E-Mail: lilimin1970@126.com; 2 Agriculture and Agri-Food Canada, Guelph Food Research Centre, Guelph, Ontario, N1G 5C9, Canada; E-Mail: wangq@agr.gc.ca

**Keywords:** β-glucan, molecular characterization, hull-less barley, flour, shorts, bran

## Abstract

Six hull-less barley cultivars widely grown in China were roller-milled to produce bran, shorts and flour fractions. The distribution and molecular characteristics of β-glucans from the three roller-milled fractions were investigated. The β-glucan contents in the six hull-less barley cultivars varied from 4.96% to 7.62%. For all the six cultivars, the shorts fraction contained the highest concentration of β-glucan (8.12–13.01%), followed by bran (6.15–7.58%) and flour (2.48–2.95%). Crude β-glucans were prepared from the three roller-milled fractions using aqueous sodium carbonate (pH 10). These preparations contained 45.38–71.41% β-glucan, 10.81–17.26% arabinoxylan, 2.6–9.6% protein, 2.7–9.0% starch, and 5.23–9.68% ash. Purification using α-amylase and β-xylanase in combination with pH adjustment and dialysis produced high purity β-glucan preparations (91–95%). The molecular weight (Mw) of β-glucan preparations from roller-milled fractions ranged from 117,600 to 852,400 g/mol. β-Glucan from flour had higher Mw than those from shorts and bran within the same cultivar, and β-glucan preparations from bran had the lowest Mw.

## 1. Introduction

Hull-less barley is the main staple food crop in Tibet, grown in more than 55% of the total cultivated land. All the cultivated varieties grown in Tibet are hull-less barley. In contrast to regular hulled barley, hull-less or naked barley offers more advantages to processing and food uses [1]. There is a long history of Tibetans preparing food products from hull-less barley in many unique ways. The main and popular products of hull-less barley are Tsangpa, a type of roasted barley flour, and Chang, a alcoholic beverage brewed from hull-less barley. In addition, barley has been used for making steamed bread, noodles, cakes, soups, and snack foods [2,3]. Hull-less barley has recently attracted a lot of interests among food scientists and technologists because of its soluble dietary fiber, which may provide considerable health benefits [4,5]. Such physiological benefits have generally been attributed to β-glucan, a group of non-starchy polysaccharides and the primary component of soluble dietary fiber in both barley and oats [4]. The amount of β-glucan in hull-less barley normally varies from 4% to 8% [6]. Levels of β-glucan are influenced by both genetic and environmental factors, but genetic factor appears to be of greater importance [7]. Barley cultivars expressing the hull-less or waxy traits generally have high β-glucan content [8]. β-Glucans are found in the cell walls of many cereals. Compared to other cereals, barley and oat have relatively higher levels of β-glucans, which range from 2% to 10% in barley and 2.5% to 6.6% in oat [9].

β-Glucan of barley and oats can be enriched by dry milling and sieving, dry milling and air classification, or prepared in high concentrations by wet extraction and purification [10,11]. Due to the beneficial physiological effects associated with water-soluble β-glucans, there has been some interest in optimizing procedures for water extractability of these polymers from barley by controlling the temperature and pH of aqueous solvents [12]. Molecular characteristics of β-glucans have been determined using high-performance size-exclusion chromatography (HPSEC) in combination with light scattering detector, refractive index and viscometric detectors [13,14]. The combination of size exclusion chromatographic profile with the result calculated from multiple detectors allows simultaneous characterization of intrinsic viscosity, radius of gyration, molecular weight and molecular weight distribution of β-glucans [15].

Hull-less barley can be easily separated into flour and bran fractions using conventional wheat-milling equipment [16]. Although barley has not traditionally been roller-milled like wheat to obtain flour and bran, this may change in the near future because of barley’s high soluble fiber content and its potential use in many food products. The objective of this study is to obtain detailed information on the distribution and molecular properties of β-glucans among six hull-less barley cultivars and their roller-milled fractions. The information obtained will provide indications of the nutritional quality and potential uses of the milled products.

## 2. Results and Discussion

### 2.1. Characteristics of Hull-Less Barley Samples

The kernel properties and chemical composition of hull-less barley varieties tested are summarized in Table 1. The main components of the hull-less barley are starch 55.4–56.9% and crude protein 9.2–10.2%. β-Glucan and arabinoxylan are the major nonstarchy polysaccharides; the β-glucan content is generally higher than arabinoxylan content from the whole kernel. β-Glucan content varies from 4.96% to 7.62%, and is in good agreement with the previous findings in which β-glucan contents were found to range from 2.4% to 8.3% in hull-less barleys [17]. Test weight and 1000 kernel weight were the indicators of kernel properties. The results showed that hull-less barley—with higher test weight and 1000 kernel weight—contained more β-glucan.

### 2.2. Yields and Chemical Compositions of Hull-Less Barley Roller-Milled Fractions

Hull-less barley was roller-milled into three fractions: bran, shorts and flour, the compositions of which are tabulated in Table 2 for the six hull-less barley samples. The yields of the bran, shorts and flour were in the range of 16.3–18.1%, 10.2–12.5% and 69.0–71.3%, respectively. Bran and shorts contained higher concentrations of ash, protein, β-glucan and arabinoxylan than flour did; whereas flour contained much higher amount of starch.

**Figure 1.**
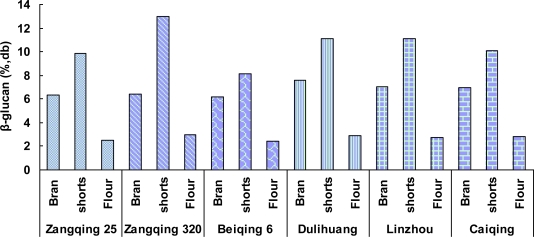
Distribution of of β-glucan in roller-milled fractions of different hull-less barley cultivars.

**Figure 2.**
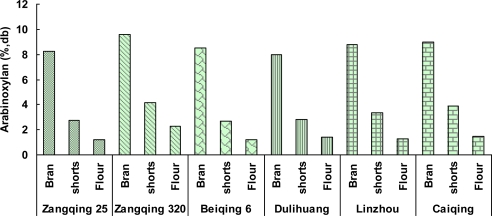
Distribution of arabinoxylan in roller-milled fractions of different hull-less barleys.

The distribution of β-glucan and arabinoxylan in the fractions were also different as evidenced in Figures 1 and 2. Shorts contained the highest concentration of β-glucan (8.12–13.01%), followed by bran (6.15–7.58%) and flour (2.48–2.95%). Similar results were obtained on milling of hull-less barley samples by Andersson *et al.* using a laboratory mill [18]. This seems to suggest that the distribution of β-glucan in the hull-less barley kernel under this investigation is uneven, mainly concentrated in the aleurone and subaleurone layers which form the major component of shorts. The bran mainly contained the outer layers of the kernel and some cell walls from the subaleurone layer. The latter contributed to the enrichment of β-glucan in bran. The flour had the lowest concentration of β-glucan, which was derived from the endosperm cell walls. Accumulating evidence has indicated that the distribution of β-glucan in barley kernel varies with varieties [19,20]. It was observed that in low β-glucan content hull-less barleys, β-glucan was concentrated in the subaleurone and endosperm immediately adjacent to subaleurone; whereas in high β-glucan content barleys, β-glucan was more uniformly distributed throughout the endosperm [19,21]. β-Glucans concentrated in the aleurone layer of barley kernel has also been reported [21,22]. Therefore, awareness of the distribution of β-glucan in the kernel may provide guidance for the production of β-glucan enriched barley products.

The distribution of arabinoxylan in hull-less barley kernel was also uneven. Bran contained the highest concentration of arabinoxylan (7.99–9.59%), followed by shorts (2.29–3.86%) and flour (1.2–2.29%). The results indicate arabinoxyaln mainly existed in the outer bran part, then the aleurone part, and the endosperm had the lowest arabinoxyaln concentration.

### 2.3. Yields and Compositions of β-Glucans from Hull-Less Barley Roller-Milled Fractions

Crude β-glucans were prepared from the three roller-milled fractions of hull-less barleys using aqueous sodium carbonate. The yield and composition of crude β-glucan extracts are listed in Table 3. For all the six barley samples, the yields of crude β-glucans followed the order of shorts (9.47–13.77%), bran (7.24–8.28%) and flour (2.83–3.44%). The purities of the β-glucans were in the range of 45–71% with the highest purity obtained from the flour extracts and the lowest from the bran extracts. There were considerable contaminations of arabinoxylan (10.8–17.3%), protein (2.6–9.6%), starch (2.7–9.0%), and ash (5.23–9.68%). Higher arabinoxylan content in the crude β-glucan preparations indicated that pH 10 aqueous sodium carbonate could also extract arabinoxylan.

The crude β-glucan preparations were further purified by applying α-amylase and β-xylanase to eliminate starch and arabinoxylan impurities. Protein was partially removed by heating and adjusting pH close to the isoelectric points of proteins. Dialysis was used to remove low molecular weight materials. High purity β-glucan preparations were obtained after such purifications (91–95%), only slightly lower than the commercial barley β-glucan standards (97–98%). The yields and compositions of purified β-glucan preparations are shown in Table 4. The arabinoxylan, starch, protein, and ash contents were reduced remarkably compared with crude β-glucan preparations, which indicated that the purification procedure employed was effective to purify crude β-glucan preparations.

### 2.4. Molecular Properties of β-glucans from Roller-Milled Fractions of Hull-Less Barleys

The molecular properties of purified β-glucan preparations from roller-milled fractions of hull-less barleys were determined by HPSEC-RALLS-DP-RI. The results are summarized in Table 5 together with the three barley β-glucan standards. Large variations in the molecular weight of purified β-glucans from different fractions within each cultivar and among the six cultivars were observed. The molecular weights ranged from 117,600 to 852,400 g/mol, which were somewhat lower than those reported in the literature (4 × 10^5^–2 × 10^6^ g/mol) [23–25].The varietal difference in molecular weight among barley has been well documented in these investigations. In our study, we noticed that the purification process caused reduction in the molecular weight of β-glucan. This may also attribute to the lower molecular weight values obtained. For the same hull-less barley cultivar, β-glucan from flour yielded the highest Mw compared to those from shorts and bran. β-Glucan preparations from bran had the lowest Mw. Barley flour seems to be a better source for the preparation of high molecular weight β-glucan standard. The molecular weights of the three β-glucan standards were found to be 370,900, 248,900 and 158,300 g/mol respectively for high, medium and low viscosity samples which fell in the lower end range of the barley β-glucan preparations.

**Figure 3.**
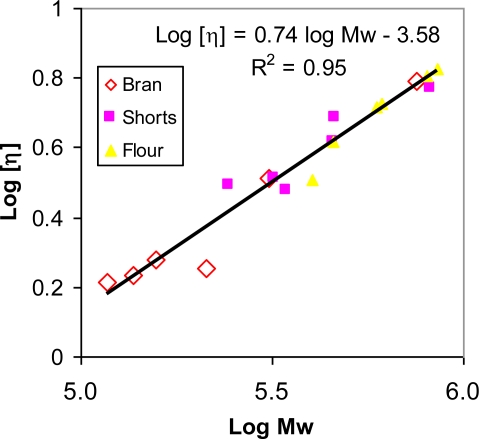
Mark-Houwink plots of β-glucans from hull-less barley roller-milled fractions.

The intrinsic viscosities of purified β-glucans from all the fractions ranged between 1.6 and 6.8 dL/g. The relationship between molecular weight and intrinsic viscosity followed a common Mark-Houwink plot (Figure 3), with an exponent of 0.74, irrespective of the source of β-glucans. This indicates that there is no significant conformational difference between β-glucans from bran, shorts and flour.

## 3. Experimental Section

### 3.1. Materials

Six hull-less barley cultivars were obtained from Lanzhou Qizheng Group (Gansu province, China) during the 2008 crop year. The barley cultivars were Zangqing 25, Zangqing 320, Beiqing 6, Linzhou, Dulihuang, and Caiqing. Thermal stable α-amylase, β-xylanase, β-glucosidase were purchased from Megazyme International Ireland Ltd (Bray, Ireland). Three barley β-glucan standards with low, medium, and high viscosity were purchased from Megazyme International Ireland Ltd (Bray, Ireland)). All chemicals were of reagent grade.

### 3.2. Hull-Less Barley Milling

The moisture of hull-less barley samples were adjusted to 16% and conditioned for 30 h at room temperature (about 25 °C). The conditioned hull-less barley was milled using a Buhler MLU 202 laboratory mill (Buhler Corp, Switzerland). The laboratory mill has three break systems and three middling systems. The six fractions from the starchy endosperm were combined to give a flour fraction. Bran and shorts fractions were also collected. Bran was ground to pass 40 mesh sieve. Whole meal flour was obtained by combination of all milling fractions.

### 3.3. Preparation and Purification of β-Glucans

β-Glucans were prepared from hull-less barley flour, shorts, and bran according to the procedure of Cui [26] with some modification. Hull-less barley flour, shorts, or bran was refluxed in 70% ethanol. The ethanol extract was separated from residue by vacuum filtration. The residue was washed with 100% ethanol, vacuum filtered, air-dried at room temperature, then vacuum dried. The ethanol refluxed sample was blended with distilled water, and adjusted to pH 10 with 20% sodium carbonate. The mixture was centrifuged (×5000 g, 10 min, 25 °C) and the supernatant (S1) was decanted. The residue was blended with aqueous sodium carbonate at pH 10 and re-extracted. The mixture was centrifuged and the supernatant (S2) was decanted. The liquid extracts (Sl and S2) were mixed together and adjusted to pH 4.5 with 6 M HCl under stirring, then kept at 25 °C for 1h without stirring. The resulting mixture was centrifuged, and added an equal volume of absolute ethanol with vigorous stirring, and retained at 4 °C overnight. The mixture was centrifuged and the supernatant was carefully decanted. The precipitate was washed with 95% ethanol, 100% ethanol, and followed by vacuum dried at 50 °C for 3 h to give crude β-glucan preparations.

The crude β-glucan preparation was dissolved in pH 10 aqueous sodium carbonate solution at 50 °C for 60 min. The solution pH was adjusted to 6.5 with 6 M HCl, then treated with thermostable α-amylase for 1 h at 90 °C to eliminate starch impurities. After cooling to room temperature, the pH was adjusted to 4.5 and the mixture was centrifuged (×5000 g, 10 min, 25 °C). The supernatant was neutralized to pH 4.75. β-Xylanase (100 unit/100 mL) in sodium acetate buffer (0.2 mol/L, pH 4.75) were added at 50 °C with constant stirring for 2 h. The enzyme was deactivated by heating in boiling water bath for 30 min. The mixture was centrifuged (×5000 g, 10 min, 25 °C). To the supernatant, an equal volume of absolute ethanol was added with vigorous stirring. The mixture was centrifuged (×5000 g, 10 min, 25 °C) after standing overnight at 4 °C. The supernatant was carefully decanted. The pellet was dissolved in distilled water by heating to 50 °C overnight with constant stirring, and cooled to room temperature. The solution was dialyzed against distilled water for 48 h. An equal volume of 100% ethanol was added slowly with vigorous stirring, and the precipitate allowed to settle, then collected by centrifugation (× 5000 g, 10 min, 25 °C). The pellet was washed with 100% IPA, and dried in the vacuum oven at 40 °C for 2 h to obtain purified β-glucan.

### 3.4. Test Weight and 1000 Kernel Weight

Test weight of hull-less barley cultivar was determined according AACC55-10 method. 1000 kernel weight of hull-less barley was measured with Perten SKCS (Perten Instrument AB, Sweden). Samples were loaded into the Perten SKCS and tested according to the operating manual procedure (Single Kernel Characterisation System 4100 Manual, 1995).

### 3.5. Chemical Analyses

Moisture and ash contents were measured by AACC methods 44-15A and 08-01, respectively. Crude protein was analyzed by NA2100 Nitrogen & Protein Analyzer using the factor of 5.7 for hull-less barley flour and 6.25 for hull-less barley shorts and bran to convert nitrogen to protein. Starch was analyzed according to standard method 76–11 (AACC 2004). Total arabinoxylan content was analyzed using the colorimetric method of Dougals [27].The β-glucan content was determined by the specific enzyme method [28] using a β-glucan assay kit supplied by Megazyme International Ireland Ltd (Bray, Ireland).

### 3.6. Characterization of β-glucan by Size Exclusion Chromatography (HPSEC)

Molecular weight, radius of gyration and intrinsic viscosity of β-glucan preparations were determined by HPSEC equipped with three detectors (HPSEC-RALLS-DP-RI, Triple Detector System, Model Dual 250, Viscotek, Houston, TX). β-Glucans were dissolved in pure water at 70 °C for 2 h, then stirred overnight at room temperature. 100 μL samples were injected to the HPSEC column after being filtered with 0.45 μm filter. The chromatographic system included a Shimadzu SCL-110Avp pump with automatic injector (Shimadzu Scientific Instruments Inc. Columbia, Maryland 21046, USA), two columns in series: a Shodex Ohpak KB-806M (Showa Denko K.K., Tokyo, Japan), and an Ultrahydrogel linear (Waters Milford, CT, USA). To test the accuracy of the calibration, three pullulan standards (P-100, P-400, and P-800) were tested. Recovery of these polysaccharide standards was >95%. The M_w_ values were 110,400 for P-100, 405,800 for P-400, and 767,000D for P-800. These values are in good agreement with those given by the manufacturer (112,000, 404,000, and 788,000).

### 3.7. Statistical Analyses

Statistical analysis of all the data was performed using Microsoft Excel.

## 4. Conclusions

Hull-less barley can be dry roller-milled with equipment routinely used in wheat milling to obtain flour (70%), shorts (10%) and bran fractions (20%). The distribution of β-glucan in hull-less barley kernel was uneven, being more concentrated in the aleurone and subalerone regions, followed by the outer layer. The endosperm had the lowest β-glucan concentration. Consequently, hull-less barley shorts contained the highest concentration of β-glucan, followed by bran and flour, indicating hull-less barley shorts is a good source of β-glucan enriched product. Crude β-glucan with 50–70% purity can be obtained from hull-less barley roller-milled fractions by extraction in aqueous solution of sodium carbonate (pH 10). Crude β-glucan preparations can be further purified to obtain higher than 90% purity. For the same hull-less barley cultivar, β-glucan from flour had higher Mw than those from shorts and bran. Thus barley flour is a better source for the preparation of high molecular weight standards.

In addition, hull-less barley bran contained higher arabinoxylan than shorts and flour, so bran and shorts can be mixed to enrich non-starchy polysaccharides, arabinoxylan and β-glucan. The observations from this study suggests that the roller-milled hull less barley fractions offer a great deal of promise as an ingredient in food, feed and industrial uses.

## Figures and Tables

**Table 1.** Characteristics of the six hull-less barley cultivars.

**Sample**	**Test weight (g/L)**	**1000 Kernel Wt (g)**	**Ash (%, db)**	**Starch (%, db)**	**Crude protein (%, db)**	**Arabinoxylan (%, db)**	**β-glucan (%, db)**
Zangqing 25	786 ± 6	51.3 ± 1.4	1.79 ± 0.01	55.4 ± 1.1	9.4 ± 0.4	4.73 ± 0.02	7.62 ± 0.01
Zangqing32	727 ± 7	39.9 ± 0.3	1.80 ± 0.00	56.9 ± 0.9	9.2 ± 0.1	3.93 ± 0.01	4.96 ± 0.03
Beiqing 6	769 ± 4	47.3 ± 1.3	1.65 ± 0.02	56.2 ± 1.4	10.1 ± 0.1	2.97 ± 0.01	6.08 ± 0.02
Dulihuan	742 ± 6	41.4 ± 0.9	1.93 ± 0.01	55.6 ± 0.7	10.1 ± 0.3	3.25 ± 0.04	5.61 ± 0.02
Lizhou	756 ± 9	43.3 ± 0.7	1.81 ± 0.00	55.9 ± 0.6	10.2 ± 0.3	3.16 ± 0.02	5.42 ± 0.01
Caiqing	737 ± 5	40.8 ± 1.0	1.87 ± 0.01	55.6 ± 1.2	10.2 ± 0.2	3.63 ± 0.03	5.29 ± 0.02

Analyses were conducted in duplicate; The mean ± standard deviation is shown; db: dry basis.

**Table 2.** Yields and chemical compositions of hull-less barley roller-milled fractions (%, dry basis).

**Sample**		**Yield**	**Ash**	**Starch**	**Protein**	**Arabinoxylan**	**β-glucan**
Zangqing 25	Bran	16.7	3.43 ± 0.01	31.1 ± 0.4	19.3 ± 0.2	8.24 ± 0.01	6.34 ± 0.02
	shorts	11.1	1.80 ± 0.00	43.8 ± 0.3	12.7 ± 0.2	2.75 ± 0.02	9.85 ± 0.03
	Flour	71.3	0.81 ± 0.01	68.8 ± 1.0	11.8 ± 0.1	1.20 ± 0.01	2.48 ± 0.01
Zangqing320	Bran	16.3	3.50 ± 0.01	28.5 ± 0.2	17.4 ± 0.2	9.59 ± 0.01	6.44 ± 0.01
	shorts	12.5	1.77 ± 0.00	41.6 ± 0.4	10.9 ± 0.1	4.18 ± 0.01	13.01 ± 0.02
	Flour	69.5	0.66 ± 0.01	67.9 ± 0.9	11.7 ± 0.1	2.29 ± 0.01	2.95 ± 0.00
Beiqing6	Bran	17.0	3.19 ± 0.00	35.8 ± 0.5	18.6 ± 0.1	8.51 ± 0.02	6.15 ± 0.00
	shorts	12.4	1.78 ± 0.01	49.8 ± 0.8	11.4 ± 0.3	2.70 ± 0.03	8.12 ± 0.01
	Flour	69.4	0.74 ± 0.02	68.0 ± 1.1	11.4 ± 0.2	1.20 ± 0.02	2.40 ± 0.04
Dulihuang	Bran	18.1	2.82 ± 0.01	34.6 ± 0.3	16.9 ± 0.1	7.99 ± 0.01	7.58 ± 0.01
	shorts	11.7	1.44 ± 0.00	44.0 ± 0.7	12.5 ± 0.3	2.82 ± 0.00	11.13 ± 0.02
	Flour	69.0	0.85 ± 0.00	69.6 ± 0.7	11.9 ± 0.4	1.44 ± 0.02	2.92 ± 0.01
Linzhou	Bran	18.0	3.24 ± 0.01	27.8 ± 0.4	16.4 ± 0.3	8.76 ± 0.03	7.05 ± 0.01
	shorts	10.2	1.52 ± 0.00	48.5 ± 0.6	9.7 ± 0.0	3.34 ± 0.03	11.12 ± 0.03
	Flour	70.7	0.99 ± 0.01	68.8 ± 1.2	12.5 ± 0.1	1.26 ± 0.02	2.72 ± 0.01
Caiqing	Bran	16.4	2.51 ± 0.02	31.5 ± 0.3	15.8 ± 0.3	9.01 ± 0.01	6.95 ± 0.02
	shorts	11.3	1.15 ± 0.02	48.1 ± 0.6	10.6 ± 0.3	3.86 ± 0.01	10.08 ± 0.01
	Flour	71.1	0.76 ± 0.01	69.1 ± 1.0	11.5 ± 0.2	1.49 ± 0.02	2.80 ± 0.01

**Table 3.** Yield and composition of crude β-glucan from hull-less barley roller-milled fractions.

**Sample**		**Yield (%)**	**Ash (%, db)**	**Starch (%, db)**	**Protein (%, db)**	**β-glucan (%, db)**	**Arabinoxylan (%, db)**
Zangqing 25	Bran	7.68 ± 0.51	9.15 ± 0.11	2.7 ± 0.5	5.6 ± 0.1	58.03 ± 0.92	16.79 ± 0.42
	shorts	10.23 ± 0.72	8.37 ± 0.07	4.3 ± 0.1	3.1 ± 0.0	65.73 ± 0.83	11.84 ± 0.53
	Flour	3.06 ± 0.14	5.23 ± 0.08	5.8 ± 0.3	3.4 ± 0.1	70.4 ± 0.81	12.86 ± 0.32
Zangqing320	Bran	7.24 ± 0.66	8.64 ± 0.12	5.3 ± 0.6	5.5 ± 0.0	45.38 ± 0.74	17.26 ± 0.23
	shorts	13.77 ± 0.77	7.79 ± 0.09	7.3 ± 0.5	2.8 ± 0.0	61.26 ± 0.62	13.59 ± 0.33
	Flour	3.18 ± 0.21	6.29 ± 0.06	2.8 ± 0.5	5.3 ± 0.1	70.04 ± 0.82	14.28 ± 0.28
Beiqing6	Bran	7.33 ± 0.33	9.32 ± 0.10	2.7 ± 0.3	7.2 ± 0.1	60.7 ± 0.88	15.76 ± 0.30
	shorts	9.47 ± 0.57	9.54 ± 0.07	5.3 ± 0.2	3.6 ± 0.2	65.82 ± 0.63	11.08 ± 0.09
	Flour	2.95 ± 0.30	7.71 ± 0.06	4.7 ± 0.2	9.6 ± 0.0	66.29 ± 0.82	10.81 ± 0.14
Dulihuang	Bran	8.28 ± 0.61	8.11 ± 0.10	3.3 ± 0.1	4.8 ± 0.1	56.15 ± 0.64	16.13 ± 0.19
	shorts	11.73 ± 0.69	9.64 ± 0.09	5.4 ± 0.1	4.0 ± 0.1	58.77 ± 0.53	13.41 ± 0.22
	Flour	3.40 ± 0.24	7.98 ± 0.07	6.2 ± 0.0	5.5 ± 0.1	61.65 ± 0.91	13.3 ± 0.25
Linzhou	Bran	8.26 ± 0.58	9.39 ± 0.08	2.9 ± 0.2	4.8 ± 0.0	58.05 ± 0.76	16.21 ± 0.13
	shorts	12.01 ± 0.97	7.47 ± 0.10	9.0 ± 0.1	3.1 ± 0.2	58.45 ± 0.82	13.03 ± 0.25
	Flour	3.44 ± 0.22	8.24 ± 0.06	3.2 ± 0.1	4.3 ± 0.0	66.57 ± 0.67	14.49 ± 0.46
Caiqing	Bran	7.91 ± 0.45	9.68 ± 0.05	3.5 ± 0.8	5.3 ± 0.1	51.61 ± 0.81	16.48 ± 0.37
	shorts	11.03 ± 0.69	8.19 ± 0.07	7.8 ± 0.4	2.6 ± 0.1	60.52 ± 0.77	12.76 ± 0.26
	Flour	2.83 ± 0.23	7.76 ± 0.09	5.1 ± 0.0	4.4 ± 0.1	71.41 ± 0.32	11.69 ± 0.22

Yield = (weight of crude β-glucan/roller-milled fraction) × 100%

**Table 4.** Yields and compositions of purified β-glucans from roller-milled barley fractions.

**Samples**		**Yield (%)**	**Ash (%, db)**	**Starch (%, db)**	**Protein (%, db)**	**β-glucan (%, db)**	**Arabinoxylan (%, db)**
Zangqing 25	Bran	4.25 ± 0.36	0.34 ± 0.02	2.3 ± 0.66	1.49 ± 0.66	91.38 ± 0.66	2.24 ± 0.66
	shorts	7.47 ± 0.42	0.49 ± 0.03	2.1 ± 0.66	2.14 ± 0.66	92.04 ± 0.66	2.47 ± 0.66
	Flour	1.84 ± 0.06	0.39 ± 0.01	2.7 ± 0.66	1.81 ± 0.66	90.05 ± 0.66	2.32 ± 0.66
Beiqing6	Bran	3.88 ± 0.28	0.64 ± 0.04	2.4 ± 0.66	1.57 ± 0.66	94.57 ± 0.66	2.38 ± 0.66
	shorts	10.32 ± 0.56	0.51 ± 0.03	1.8 ± 0.66	1.79 ± 0.66	93.38 ± 0.66	2.74 ± 0.66
	Flour	1.72 ± 0.08	0.49 ± 0.01	2.1 ± 0.66	2.14 ± 0.66	90.88 ± 0.66	2.17 ± 0.66
Dulihuang	Bran	4.23 ± 0.32	0.34 ± 0.02	1.3 ± 0.66	2.19 ± 0.66	94.38 ± 0.66	1.24 ± 0.66
	shorts	6.58 ± 0.44	0.29 ± 0.01	2.2 ± 0.66	1.04 ± 0.66	95.04 ± 0.66	2.47 ± 0.66
	Flour	1.32 ± 0.04	0.74 ± 0.04	2.1 ± 0.66	1.69 ± 0.66	92.38 ± 0.66	3.14 ± 0.66
Linzhou	Bran	5.30 ± 0.66	0.59 ± 0.05	2.0 ± 0.66	2.04 ± 0.66	94.29 ± 0.66	2.07 ± 0.66
	shorts	8.79 ± 0.62	0.34 ± 0.02	2.0 ± 0.66	1.19 ± 0.66	92.38 ± 0.66	2.24 ± 0.66
	Flour	1.72 ± 0.02	0.33 ± 0.01	2.2 ± 0.66	1.34 ± 0.66	90.04 ± 0.66	1.40 ± 0.66
Zangqing320	Bran	5.22 ± 0.38	0.24 ± 0.02	2.3 ± 0.66	1.49 ± 0.66	95.38 ± 0.66	2.24 ± 0.66
	shorts	8.88 ± 0.64	0.49 ± 0.03	2.1 ± 0.66	1.14 ± 0.66	93.04 ± 0.66	1.47 ± 0.66
	Flour	1.58 ± 0.06	0.39 ± 0.03	2.7 ± 0.66	2.81 ± 0.66	92.05 ± 0.66	2.30 ± 0.66
Caiqing	Bran	4.49 ± 0.38	0.34 ± 0.02	2.4 ± 0.66	2.57 ± 0.66	92.57 ± 0.66	2.38 ± 0.66
	shorts	8.62 ± 0.60	0.52 ± 0.04	1.8 ± 0.66	2.79 ± 0.66	93.38 ± 0.66	2.74 ± 0.66
	Flour	1.73 ± 0.04	0.44 ± 0.02	2.1 ± 0.66	1.14 ± 0.66	94.88 ± 0.66	2.17 ± 0.66
Standard 1			0.21 ± 0.01	1.1 ± 0.66	0.54 ± 0.66	97.04 ± 0.66	0.87 ± 0.66
Standard 2			0.16 ± 0.01	0.9 ± 0.66	0.32 ± 0.66	98.04 ± 0.66	0.62 ± 0.66
Standard 3			0.25 ± 0.03	0.7 ± 0.66	0.27 ± 0.66	98.04 ± 0.66	0.47 ± 0.66

Yield = (weight of purified β-glucan/roller-milled fraction) × 100%

**Table 5.** Molecular properties of β-glucans from roller-milled fractions of hull-less barleys.

**Samples**		**Mw(×10^5^) (g/mol)**	**[η] (dl/g)**	**Rg (nm)**	**Pd (Mn/Mw)**
Zangqing 25	Bran	2.12 ± 0.02	1.80 ± 0.06	22.06 ± 0.42	2.91 ± 0.03
	shorts	3.44 ± 0.06	2.99 ± 0.08	31.25 ± 0.53	2.04 ± 0.02
	Flour	4.03 ± 0.03	3.23 ± 0.06	33.32 ± 0.38	1.27 ± 0.01
Beiqing6	Bran	1.57 ± 0.02	1.91 ± 0.02	20.25 ± 0.22	1.19 ± 0.03
	shorts	3.36 ± 0.07	3.80 ± 0.07	33.59 ± 0.65	2.17 ± 0.04
	Flour	4.55 ± 0.07	4.14 ± 0.10	39.65 ± 0.72	1.70 ± 0.01
Dulihuang	Bran	7.55 ± 0.09	6.19 ± 0.08	51.47 ± 0.68	2.10 ± 0.04
	shorts	8.23 ± 0.06	5.90 ± 0.06	53.15 ± 0.83	2.39 ± 0.03
	Flour	8.52 ± 0.07	6.73 ± 0.07	56.33 ± 1.02	1.07 ± 0.03
Linzhou	Bran	3.10 ± 0.05	3.24 ± 0.03	30.90 ± 0.77	1.91 ± 0.01
	shorts	4.62 ± 0.04	4.86 ± 0.05	41.41 ± 0.92	2.77 ± 0.02
	Flour	6.10 ± 0.06	5.32 ± 0.04	48.69 ± 1.02	1.08 ± 0.02
Zangqing320	Bran	1.37 ± 0.02	1.71 ± 0.01	19.20 ± 0.38	1.83 ± 0.05
	shorts	2.43 ± 0.03	3.11 ± 0.03	28.34 ± 0.42	1.74 ± 0.04
	Flour	5.91 ± 0.07	5.19 ± 0.07	45.42 ± 0.76	1.37 ± 0.03
Caiqing	Bran	1.18 ± 0.02	1.63 ± 0.03	17.85 ± 0.23	2.03 ± 0.03
	shorts	3.19 ± 0.05	3.26 ± 0.04	33.44 ± 0.54	1.89 ± 0.05
	Flour	8.05 ± 0.09	6.43 ± 0.09	54.63 ± 0.68	1.78 ± 0.06
Standard 1	High viscosity	3.71 ± 0.03	4.15 ± 0.05	37.16 ± 0.62	1.26 ± 0.02
Standard 2	Medium viscosity	2.49 ± 0.05	2.99 ± 0.03	29.10 ± 0.32	1.34 ± 0.02
Standard 3	Low viscosity	1.58 ± 0.01	2.53 ± 0.03	23.86 ± 0.44	1.19 ± 0.04

Mw = weight average molecular weight; Mn = number-average molecular weight;

Rg = radius of gyration; [η] = intrinsic viscosity; Pd = M_w_/M_n_.
